# A Case for Case Reports: How to Write One and Promote Mentorship, Scholarship and Faculty Development

**DOI:** 10.7759/cureus.33299

**Published:** 2023-01-03

**Authors:** Shanu Gupta, Alyssa Kimble, Tracey Henry, Alfred Burger, Rahul Mhaskar, Lauren Block

**Affiliations:** 1 Internal Medicine, University of South Florida, Tampa, USA; 2 Internal Medicine, Emory University School of Medicine, Atlanta, USA; 3 Internal Medicine, Mount Sinai Beth Israel, New York City, USA; 4 Internal Medicine, University of South Florida Morsani College of Medicine, Tampa, USA; 5 Internal Medicine, Northwell Hofstra, New York, USA

**Keywords:** interactive workshop, academic rank, gme scholarly activity, mentorship program, faculty development in medical education

## Abstract

Introduction

Case reports form the base layer of the evidence pyramid, describing new or emerging diseases, side effects to treatments, common presentations of rare diseases, or rare presentations of common diseases. An important scholarly pursuit, writing case reports can be hindered by lack of time, training, and mentorship. Here, we describe a workshop incorporating case writing skills with mentorship opportunities to engage faculty and learners.

Methods

We designed and implemented a virtual, synchronous workshop addressing knowledge and attitudes on case reports for trainees and academic faculty at distributed sites. Participants discussed the contributions of case reports to the medical literature, key features of successful cases, approaches to writing learning objectives, and how to develop interesting cases into dynamic case reports. Case reports were discussed as a way to mentor learners to disseminate interesting cases as a source of clinical experience and academic productivity.

A retrospective pre-post survey was collected two months after the workshop to evaluate its utility.

Results

Fifteen out of 42 participants responded to the survey. As a result of the workshop, respondents noted improvement in confidence in identifying and writing case reports and identifying and working with mentors or mentees, regardless of level of training or specialty. At the follow-up, seven (47%) respondents had identified a case and 10 (67%) had identified a mentor/mentee to write a case report with.

Discussion

This workshop, successfully delivered virtually, demonstrates the utility of a brief educational intervention in improving participant confidence in identifying and writing case reports with mentorship.

## Introduction

Writing case reports (CRs) can offer value in optimizing quality and timely patient care and an avenue for research productivity and mentoring within an academic setting. Case reports allow healthcare professionals to develop and update illness scripts and clinical reasoning skills. Case reports allow for rapid publication on emerging diseases, most recently providing a key source of clinical understanding early in the COVID-19 pandemic. Case reports and case series continue to form the base layer of the evidence pyramid and provide dissemination of information detailing side effects to treatments; common presentations of rare diseases; or rare presentations of common diseases [1,2]. For authors, CRs can be a viable source of academic recognition for students and faculty through oral or poster presentations at academic conferences. Writing CRs offers several skills valuable in research. Authors must do a thorough literature review and use clear and concise scientific writing, including learning objectives and take-home points [3,4]. Case reports can be published in peer-reviewed journals, contributing to scholarship. For clinical faculty and trainees, scholarly activity is expected, yet clinical productivity limits the time available to dedicate to research projects [5,6]. Case reports may provide an approachable avenue for scholarship for the busy clinician while satisfying intellectual curiosity and fulfilling the requirements of governing bodies for academic training programs.

Although content development for a CR may be considered less burdensome than for more extensive research projects, several challenges have been noted in the literature. Barriers to writing CRs include lack of time, funding, experience, and mentorship [7,8]. Curricular interventions have demonstrated lasting outcomes with increased scholarly output at the residency level, but these have been primarily longitudinal and single-institution in nature [9-11]. Single workshops have demonstrated an improvement in learner self-perceptions of competence in writing and submitting CRs [12]. Additional workshops and longitudinal curricula have demonstrated success in developing research mentoring relationships without long-term follow-up on mentoring outcomes [13,14].

Our aim was to empower clinicians and inspire scholarship and mentorship together with tangible long-term outcomes. To better support clinicians in this endeavor, we developed a synchronous, remote workshop to teach residents, fellows, and faculty members the skills and format involved in writing CRs. To engage clinician educators, we included a focus on mentoring trainees through the process of selecting, writing, and submitting CRs. This approach encouraged participants with varying research backgrounds to share their experiences, perspective, and knowledge, and view CRs as an opportunity for research and mentorship, and dissemination of clinical findings.

## Materials and methods

Setting and participants

This workshop was developed and delivered by a facilitator group drawn from four institutions (SG, AK, TH, AB, LB). The authors have published CRs with experience in writing and mentoring junior learners through the writing and publishing process. The authors (SG, TH, AB, LB) serve as associate editors for the newsletter for the Society of General Internal Medicine, SGIM Forum, which features a Morning Report column where cases are reported demonstrating clinical reasoning in practice.

Our target audience included clinician educators of all specialties, at all career levels. The workshop was delivered virtually at two institutions in 2021. In the first workshop delivery, the audience consisted of 10 General Internal Medicine (GIM) fellows and four GIM faculty at an academic medical center in New York (site 1). The second workshop was delivered to 32 faculty, residents, and non-clinical staff with roles in graduate medical education (GME) associated with a GME consortium in Florida (site 2). The audience was not required to have any prior knowledge of the process of identifying, writing, or publishing CRs. The workshop was delivered on Zoom (Banyai, Istvan. (1995). Zoom. New York: Viking) with an initial didactic with subsequent small breakout rooms, facilitated by the authors.

Program description

The 90-minute workshop included a mini-didactic and two small group interactive breakout sessions (Appendix A). Participants were asked to share their roles and experiences with writing CRs and mentoring learners through writing CRs verbally and through a virtual poll. Topics covered in the 20-minute mini-didactic included a historical perspective on CRs, evidence of students and residents requesting mentoring on writing CRs, an approach to formatting CRs, and a discussion of ethical implications, including anonymity and informed consent. Finally, avenues were discussed for submitting CRs, including peer-reviewed journals.

Small group breakouts were led by workshop facilitators for 15 minutes each followed by a 10-minute debrief. The first breakout focused on identifying a CR topic, selecting novel topics about that case, and approaching learners or mentors to help write the CR together. Groups shared ideas during a large group debrief, followed by a discussion on what makes CRs novel, including common cases with unique features or learning points, uncommon cases illustrating a more universal theme, adverse reactions to treatment, emerging diseases, risk factors, or new treatments, or cases veering from the commonly held viewpoint. 

The second breakout engaged participants to write learning objectives based on a CR written by one of the facilitators. Groups shared their learning objectives during a second large group debrief followed by a discussion of relevant literature searches, what makes a case unique, and which diagnostic data and imaging to include.

The workshop wrap-up included how CRs benefit trainees, faculty, readers, and ultimately patients. Participants were encouraged to ask questions and share take-home points.

## Results

Session evaluation focused on participant-reported knowledge, attitudes, and behaviors in writing CRs. At the end of the workshops, participants were asked to discuss their learning and provide feedback to the facilitators. Two months after the workshop, in a retrospective pre-post questionnaire, participants were asked to rate their confidence in writing CRs and mentoring learners before and after the workshop, and how many CRs they had identified, discussed with a learner, written, and submitted before and after the workshop. All participants provided survey consent to participate. Data were collected anonymously. We report frequencies for the categorical data. The differences in participants’ confidence regarding writing a CR and mentoring a junior faculty member before and after the educational session were investigated by the Wilcoxon signed rank test. The differences in change in confidence (for writing a CR and mentoring) after the workshop based on participants’ rank (attending vs. fellow, etc.), medical specialty (emergency medicine vs. internal medicine, etc.) and the specific workshop attended (Site 2 vs. Site 1) was assessed by Chi-square or Fisher's exact test. P values less than 0.05 indicated statistical significance. Statistical analysis was conducted using PSS version 26 software. This evaluation was exempted by the Institutional Review Board (IRB).

The two workshops were attended by 42 participants, including six learners, 26 clinical faculty, and 10 non-clinical staff. Of these, 22 participants (52%) responded to the end-of-session evaluation - 10 from the first workshop and 12 from the second. Additionally, 15 participants (36%) responded to the retrospective pre-post survey, with 10 participants from the first workshop, four from the second workshop, and one unidentified. The respondents represented Neonatal-Perinatal Medicine, Internal Medicine, Pediatrics, Emergency Medicine, Addiction Medicine, and Obstetrics and Gynecology specialties. Nonclinical participants included librarians and research project coordinators.

Prior to the workshop, 6/15 (40%) participants reported experience with publishing CRs (Table 1). Following the workshop, 12 out of 15 (80%) respondents reported an improvement in confidence in writing a CR (p=0.002) (Table 2). Similarly, 13 of 15 (87%) respondents reported an increase in confidence in mentoring a junior learner (p=0.001). Those that reported no change in confidence were more likely to have published a CR prior to the workshop. Subgroup analysis demonstrated no significant difference in confidence levels between specialties (p=0.15) or professional backgrounds (p=0.86). Subsequent to the workshop, seven respondents reported having at least identified a case to report, three had written an abstract, and two had submitted a CR. Additionally, eight respondents reported having identified a mentor or mentee to work with on a CR since the workshop.

**Table 1:** Pre-Post Survey Responses USF: University of South Florida

Q1	Q2	Q3	Q4	Q5	Q6	Q7	Q8	Q9	Q10	Q11	Q12	Q13	Q14
Are you a... - Selected Choice	Are you a... - Other (please elaborate) - Text	What is your specialty?	Which workshop did you attend?	BEFORE attending the workshop, had you published a case report?	BEFORE you attended the workshop, how confident did you feel in writing a case report?	AFTER having attended the workshop, how confident do you feel in writing a case report?	BEFORE you attended the workshop, how confident did you feel in mentoring a junior learner in writing a case report?	AFTER having attended the workshop, how confident do you feel in mentoring a junior learner in writing a case report?	Since the workshop, have you identified a case to publish?	Since the workshop, have you written an abstract for a case report?	Since the workshop, have you submitted a case report?	Since the workshop, have you identified a mentee or mentor to work with on a case report?	Since the workshop, have you worked with a mentee or mentor to submit a case report?
Attending				Yes	2	Somewhat confident	Fairly confident	Fairly confident	No	No	Yes	Yes	Yes
Attending			January 2021 at USF	Yes	Fairly confident	Fairly confident	Fairly confident	Fairly confident	No	No	No	No	No
Attending		Neonatal-Perinatal Medicine	January 2021 at USF	Yes	Slightly confident	Fairly confident	Slightly confident	Fairly confident	Yes	Yes	No	Yes	Yes
Attending		Internal Medicine	November 2020 at Northwell	Yes	Somewhat confident	Fairly confident	Somewhat confident	Fairly confident	Yes	No	No	No	No
Fellow		Internal Medicine	November 2020 at Northwell	No	Somewhat confident	Fairly confident	Slightly confident	Somewhat confident	No	No	No	Yes	No
Fellow		General Internal Medicine	November 2020 at Northwell	Yes	Somewhat confident	Fairly confident	Slightly confident	Fairly confident	Yes	No	No	Yes	No
Attending		Peds	November 2020 at Northwell	No	Fairly confident	Fairly confident	Somewhat confident	Fairly confident	No	No	No	No	No
Other (please elaborate)	GME Research Project Coordinator	NA	January 2021 at USF	No	Fairly confident	Completely confident	Fairly confident	Completely confident	No	No	No	No	Yes
Other (please elaborate)	GME Librarian	All Medical	January 2021 at USF	No	Fairly confident	Completely confident	Slightly confident	Completely confident	No	Yes	No	Yes	No
Attending		Emergency Medicine	January 2021 at USF	No	Not at all confident	Somewhat confident	Not at all confident	Slightly confident	Yes	Yes	No	Yes	No
Other (please elaborate)	Fellowship Program Director	Addiction	January 2021 at USF	No	Not at all confident	Fairly confident	Not at all confident	Somewhat confident	Yes	No	No	Yes	Yes
Attending		Obstetrics & Gynecology	January 2021 at USF	No	Not at all confident	Fairly confident	Slightly confident	Fairly confident	No	No	No	No	No
Other (please elaborate)	Research Coordinator		January 2021 at USF	No	Somewhat confident	Fairly confident	Slightly confident	Somewhat confident	No	No	No	Yes	Yes
Other (please elaborate)	Program Director	Addiction medicine	January 2021 at USF	No	Not at all confident	Somewhat confident	Not at all confident	Somewhat confident	Yes	No	No	Yes	No
Attending			January 2021 at USF	Yes	Somewhat confident	Fairly confident	Fairly confident	Completely confident	Yes	No	Yes	Yes	Yes

**Table 2:** Mean confidence levels before and after workshop delivery SD: standard deviation

	BEFORE workshop	AFTER workshop	
Writing a Case Report	2.80 (SD 1.94) N=15	3.93 (SD 0.57) N=15	P=0.002
Mentoring a Junior Learner in Writing a Case Report	2.47 (SD 1.09) N=15	3.80 (SD 0.83) N=15	p=0.001
Confidence level measured on Likert scale where 1=not at all confident, 5=completely confident			

Twenty-two participants provided free text responses to the end-of-session evaluation describing their takeaway from the workshop. Themes included opportunities for mentorship, identifying an appropriate case, identifying learning objectives, and considering progressing an abstract to publication. Table 3 shows sample responses.

**Table 3:** End-of-session evaluation responses on workshop learning

Theme	Sample response
Mentorship opportunities	“It’s ok not to be the primary caregiver or an expert in the case. You can influence and help other learners/providers.”
	“Can work with learners who weren't directly involved in a case”
Identifying appropriate case	“Think broadly of what can be used for case report and how many cases can be utilized for learning”
	“Using QI events”
	“It's pretty easy to find cases in daily practice”
Learning objectives	“To focus on the teaching point of a given case”
	“Thinking of objectives for what would catch someone’s attention”
Progression of scholarship	“Abstracts can be converted to case reports”
	“How to find appropriate target journals”
	“Being able to identify journals that fit a case”

Finally, 22 respondents in the end-of-session evaluation noted positive qualitative feedback (e.g., “Well structured, good pacing, helpful worksheet”). Suggestions included increasing the time in small group discussions and providing examples of well-written and poorly written cases.

## Discussion

Barriers to writing CRs include lack of time, mentoring, knowledge of ways to select a topic, format, and write CRs, and awareness of avenues to submit and publish CRs [7,8]. Our audience similarly noted time and lack of research and writing skills as important impediments to mentoring junior learners through scholarly activity (Table 4). Following this interactive, virtual workshop focused on skills key to writing CRs and mentoring junior learners, participants reported increased confidence in their ability to identify a case to report and a mentor or mentee. Notably, several respondents from this one-time 90-minute workshop went on to write and submit cases along with mentors/mentees in the two-month period between workshop delivery and survey follow-up. Indeed, an increase in confidence level was more likely to result in positive actions. Of those that reported a positive change in confidence (12/14), 58% (7) respondents reported having at least identified a case to report since the workshop, three (25%) having written an abstract, and two (17%) submitted a CR. Similarly, an increase in confidence in working with a mentor or mentee (13/15 respondents) resulted in positive outcomes, where eight (53%) respondents reported having identified a mentor or mentee to work with on a CR since the workshop. This suggests that a brief workshop intervention can successfully provide the impetus necessary to delve into scholarship, by bridging the lack of time and skill gap.

**Table 4:** Barriers to working with junior learners. Respondents could choose multiple responses. Participants polled during the workshop (N=22)

Barrier	% (n)
Lack of time for the learner	31% (14)
Lack of time for me	24% (11)
Lack of research skills on my part	22% (10)
Lack of writing skills on my part	7% (3)
Lack of funds	9% (4)
Lack of a mentor for me	7% (3)
Other (please comment below)	0% (0)
Total responses	100% (45)

The benefits of the workshop included a presentation at distinct institutions in different areas and applicability to a range of participants from trainees to seasoned clinical faculty and nonclinical staff. The interactive, remote workshop allowed more experienced participants to share lessons learned with the group, and the virtual platform provided access to a distributed audience and inter-institutional interaction. Inclusion of a written CR for participants to edit allowed participants to read and work with an existing piece to develop specific skills. Subsequently, the follow-up survey indicated 20% of respondents reported had written and 13% had submitted a CR.

Developing a culture of mentorship has far-reaching consequences in academic medicine [15]. Mentoring cultivates productivity and personal and career satisfaction [16]. The focus on mentoring in this workshop fostered a collaborative, inclusive approach designed to help participants succeed in starting and furthering their writing, with 53% of respondents identifying a mentor or mentee in the follow-up survey. Emblematic of the process, the author team included a resident. Upon conclusion of the workshop, the author felt empowered and subsequently sought out a research mentor, triangulating the findings of the workshop.

Limitations included a small sample size at two institutions and the use of a retrospective pre-post methodology with a low response rate. The two-month post-workshop timing was designed to assess longitudinal retention but may not have been enough time to see behavior change in terms of writing and publishing CRs. Other threats to validity include social desirability bias.

Learning communities in the form of a writing group have demonstrated success in increasing scholarly output [17,18]. In future iterations, this workshop could be supplemented with continued and formalized writing accountability groups. Additionally, the authors or other facilitators may offer one-to-one mentorship to junior learners from inception to submission of a CR.

## Conclusions

Case reports offer an opportunity for trainees and faculty to share clinical lessons learned with the medical community, improving clinical care while contributing to academic productivity. A mentoring approach to writing CRs allows a focus on collaboration. As evidenced by the responses, CRs can be a ubiquitous teaching and learning experience at all levels of training and in all specialties. By clarifying the process of selecting a case, crafting, and disseminating CRs, we encouraged participants to begin their own journey in writing and publishing CRs.

## Appendices

A Case for case reports: how to write one and promote mentorship, scholarship, and faculty development facilitator guide

Session Timeline:

**Table 5:** Suggested session timeline

Session Time	Content
00:00-00:05	Speakers and topic introduction
00:05-00:08	Getting to know the audience - audience live discussion/poll
00:08-00:12	Historical Perspective: the value of Clinical Vignettes and Case Reports then and now - didactic
00:12-00:15	Introduction to mentoring junior learners - audience live discussion/poll and didactic
00:15-00:20	Case format, Ethical concerns, Introduction to SGIM Forum Morning Report - didactic
00:20-00:25	Avenues for submitting Vignettes and Case Reports - didactic
00:25-00:40	Small group breakouts - identifying appropriate cases and sharing best practices
00:40-00:50	Large group check-in, and introduction to learning objectives - discussion and didactic
00:50-01:10	Small group breakouts - learning objectives for a pre-identified case
01:10-01:20	Large group check-in - sharing learnings from small groups
01:20-01:30	Concluding remarks and workshop evaluation

A Suggested Format for Case Reports:

**Table 6:** Suggested format for Case Reports

Abstract	< 150 words
Introduction	Overview of the problem or finding
Case	Description, history, physical exam, labs and/or radiology, treatment plan, the outcome of the case
Discussion	Include literature review Summary of salient features of the case, why they are noteworthy, how the problem is illustrated and contextualized within the existing literature
Conclusion	Key take-home points
References	< 15

Considerations for Publication of Case Reports:

**Table 7:** Considerations for Publication of Case Report

Consider the purpose of your case report.
Is this an uncommon case illustrating a more universal theme?
Does this case demonstrate an adverse reaction to treatment?
Does the case highlight an emerging disease or risk factor or new treatment?
Many “generalist” and specialist journals accept case reports based on the criteria above.
Additionally, a useful tool to identify an appropriate journal: https://jane.biosemantics.org/

Small Group Breakout Session 1:

**Table 8:** Small Group Breakout Session #1

	Start with a case
1.	Case identification. Consider a recent case you’ve encountered that you’d like to write about. What about the case would be interesting for a reader? Facilitators, consider starting the conversation with these questions: Have you already identified a case? If so, what was interesting or novel about that case? If not, how would you identify a case?
2.	Learner identification. Consider learners you have worked with. Who might you engage to help you in writing this case? Facilitators, consider starting the conversation with these questions: Can you think of a learner who you might partner with? How would you start the conversation with this learner? What challenges concern you about working with a junior learner? What might be some ways to mitigate those challenges? How can we (the presenters) support you in these?

Small Group Breakout Session 2:

**Table 9:** Small Group Breakout Session #2 PCP: primary care physician; PMH: past medical history; EGD: esophagogastroduodenoscopy; ED: emergency department; iPTH: intact parathyroid hormone

	Develop learning objectives
3.	Read the following case, and consider what learning objectives you might use to write this as a case report
	A 69-year-old woman of Haitian descent with no family history of malignancy presented to her PCP with 20-lb weight loss over eight months. She reported reflux, epigastric pain, abdominal fullness, and bloating for years. PMH included hypothyroidism on levothyroxine. She had no history of tobacco or illicit drug use. Her last mammogram and colonoscopy were normal over 10 years ago. The previous workup included bloodwork that revealed elevated alkaline phosphatase and calcium and an EGD which was normal. CT revealed soft tissue nodules throughout the lungs, supraclavicular, and mediastinal lymph nodes extending to the left mainstem bronchus, and enhancing lesions in the liver, spleen, and kidneys concerning for metastasis. She presented to a new PCP, where a physical exam was unremarkable but bloodwork revealed Ca (12.6 mg/dL). She was referred to the ED. In the ED, repeat Ca was 14.6 mg/dL and she was treated with IV fluids, furosemide, and zoledronate. Workup revealed high vitamin D 1,25 (187.8 pg/mL), normal vitamin D 25-OH (46.6 ng/mL), and iPTH (5 pg/mL, low). PTH-related peptide, SPEP, vitamin A, HIV, HBV, HCV, and HTLV were negative, and cancer antigen 125 and cancer antigen 19-9 were not elevated. A biopsy of an enlarged supraclavicular lymph node was performed and she was discharged once her calcium levels normalized with pulmonology follow-up and a presumptive diagnosis of malignancy. Following discharge, lymph node biopsy returned showing non-necrotizing granulomatous inflammation. She was seen back in primary care where her calcium level was stable and the ACE level was 110 U/L. She was referred for a liver biopsy which showed non-caseating granulomatous inflammation. Mycobacterium tuberculosis stains and histoplasmosis stains were both negative and there was no identifiable drug to support a drug-induced etiology. She was diagnosed with sarcoidosis by her pulmonologist and started on prednisone. She and her husband felt very relieved to hear she most likely did not have cancer after multiple doctors had given her a presumptive diagnosis of malignancy. After starting prednisone her appetite normalized and her weight loss, bloating, and other gastrointestinal symptoms improved. She was scheduled for a follow-up with a sarcoid specialist and repeat imaging after 6 months of treatment.
4.	Learning objectives
	Construct one or two learning objectives that would provide a scaffold to write this case. Remember to use the framework “Who will do how much of what by when.” Learning objectives should draw the reader or listener to your presentation. Use descriptive verbs - Bloom’s taxonomy is often used to identify appropriate verbs. Ideally, the learning objectives should extend beyond the specific case or diagnosis so that the learning is transferable for the learner. By the end of this case report (when), readers (who) will be able to: Facilitators, please consider starting the conversation with these questions: What would make this a case worth sharing? What LOs would you develop based on this case? Are there any non-medical knowledge-based LOs you might consider? For example, identifying biases in clinical reasoning that might have affected the premature diagnosis of malignancy. What labs or imaging would you want to include? What literature would be helpful to search?

**Table 10:** Additional Literature

Articles
Guidelines to writing a clinical case report. Heart Views. 2017;18(3):104-5.
Nissen T, Wynn R. The history of the case report: a selective review. JRSM Open. 2014.
Vandenbroucke JP. In defense of case reports and case series. Ann Intern Med. 2001;134:330-4.
Jha P, Thakur A, Klumb J, Bhandari S. Perceptions of fourth-year medical students on writing and presenting case reports. Cureus. 2018;10(3):e2341. doi:10.7759/cureus.2341.
Rivera JA, Levine RB, Wright SM. Completing a scholarly project during residency training. Perspectives of residents who have been successful. J Gen Intern Med. 2005 Apr;20(4):366-9. doi: 10.1111/j.1525-1497.2005.04157.x. PMID: 15857496; PMCID: PMC1490090.
Takahashi O et al. Residents’ experience of scholarly activities is associated with higher satisfaction with residency training. J Gen Intern Med. 2009;24(6):716-20.
Rison RA, Shepphird JK, Kidd MR. How to choose the best journal for your case report [published correction appears in J Med Case Rep. 2017 Oct 5;11(1):287]. J Med Case Rep. 2017;11(1):198. Published 2017 Jul 22. doi:10.1186/s13256-017-1351-y
